# Residential treatment exclusively for smoking cessation in patients with Crohn’s disease: Results from a pilot study

**DOI:** 10.18332/tid/149481

**Published:** 2022-06-23

**Authors:** Jens A. Leifert, Cornelia Schulz, Uta Engler

**Affiliations:** 1 Comprehensive Cancer Center Freiburg, Universitätsklinikum Freiburg, Freiburg, Germany; 2 Department of Oncology, Breisgau Klinik, Bad Kozingen, Germany

**Keywords:** residential treatment, smoking cessation, pilot study, Crohn’s disease

## Abstract

**INTRODUCTION:**

Cigarette smoking is a risk factor for the induction and severity of the course of Crohn’s disease (CD). Hospital admission may be required for treatment of the disease but is generally not available solely for smoking cessation. Outpatient group therapy is readily available, however long-term quit rates are limited. Residential treatment for smoking cessation may offer a more intense contact between patient and therapist, and may result in higher abstinence rates in a sensitive group of patients. The objective of this pilot study is to evaluate the feasibility of implementing a residential program with hospital admission, exclusively for smoking cessation for patients suffering from CD.

**METHODS:**

Twelve eligible smokers suffering from CD were recruited for a 9-day inpatient smoking cessation treatment. Treatment consisted of single and group behavioral therapy together with supportive measures such as exercise therapy, relaxation techniques or nutritional counselling. Nicotine replacement therapy or prescription medication was offered according to the Fagerström test for nicotine dependence (FTND) score and treatment guidelines. Quit rates were assessed by CO-testing during hospital treatment and by follow-up calls 6 months after discharge.

**RESULTS:**

All recruited participants arrived on time for treatment and collectively stopped smoking on the 2nd day after admission. All participants completed the therapy process without relapse and left the hospital smoke-free (100% quit rate on discharge, CO monitored). Self-reported abstinence rates after 6 months were 72.7% for continuous abstinence and 81.8% for 7-day point prevalence abstinence.

**CONCLUSIONS:**

Residential treatment exclusively for smoking cessation is feasible and efficient and may be a valuable treatment option for patients suffering from CD.

## INTRODUCTION

For patients with Crohn’s disease (CD), tobacco smoking is the most important modifiable environmental factor for the course of the inflammatory disease. Cigarette smoke is harmful to the gastrointestinal tract with negative effects on microbiota, mucosa composition or the immune system^1^. Active smokers are more likely to develop complications during the course of the disease, require intestinal surgery, have a higher recurrence rate or use immunosuppressive medication^2^. On the other hand, patients who stop smoking have a reduced risk of flare-ups or reoperation for recurrence with less use of maintenance therapy compared to continuing smokers^3^. Therefore, all CD patients who are still smoking should be strongly encouraged to quit.

Smoking cessation therapies include many therapeutic options, however, evidence for long-term effectiveness is relatively little. Even in effective (usually outpatient) group behavior therapy programs for smoking cessation, only up to 20% of smokers achieve abstinence over a period of at least 6 months^4^.

More promising results are available from studies evaluating the effects of residential treatment for patients admitted to the hospital exclusively for smoking cessation^5^ with reported quit rates between 31.0% and 64.7% after 6–12 months. In a large American cohort study, Hays et al.^6^ reported a cessation rate of 52% in 226 hospitalized patients during an 8-day inpatient treatment compared to only 27% of 4327 patients treated by the same team in an outpatient setting. In a small prospective pilot study, our group reported cessation rates of 60% (7-day point prevalence) following a 9-day residential treatment^7^.

Low outcomes in smoking cessation therapy may of course affect CD patients just like smokers in the general population. Therefore, the development of strategies to improve therapy efficiency in CD patients who smoke is important. To our knowledge, no study has evaluated the effects of a residential therapy in CD patients admitted to a hospital solely for the purpose of smoking cessation.

## METHODS

Twelve patients with Crohn’s disease and a minimum consumption of 10 cigarettes per day were recruited for a 9-day inpatient smoking cessation treatment program at the Breisgau-Klinik in Bad Krozingen, Germany. Treatment consisted of single and group behavioral therapy carried out by a team of medical doctors, psychologists or educators specifically trained for smoking cessation therapy and motivational interviewing. Patients received cessation therapy along with supportive measures such as exercise therapy, relaxation techniques or nutritional counselling. Nicotine replacement therapy (NRT) and/or prescription medication was optional to all patients according to FTND score^8^ and German treatment guidelines^9^ at their own expense. Quit rates were assessed by CO-testing during hospital treatment on a daily basis and by follow-up calls six months after discharge. Body weight was assessed by self-report before therapy and after 6 months.

## RESULTS

All twelve patients (Table 1) collectively stopped smoking on day 2 after hospital admission, completed the program without relapse and left the hospital smoke-free (100% quit rate on discharge, CO controlled). For the follow-up calls at six months, we reached 11 of the 12 patients. Unfortunately, one patient had died from lung cancer four months after discharge and had to be excluded from the evaluation, although being continuously smoke-free as reported by relatives. Self-reported rates after 6 months were 72.7% (8/11) for continuous abstinence, and 81.8% (9/11) for 7-day point prevalence abstinence; 9 of the 11 patients used NRT [patch (7), gum (1), both (1)] for a median duration of 53.1 days (range: 1–150). Surprisingly, none of the patients used prescription medication (bupropion or varenicline), although offered and even prescribed. During the 6 months post therapy period none of the patients reported having contacted other therapists or helplines but all (n=11) of the patients reported having contacted at least one of the other participants of the study. We evaluated a median self-reported weight gain of 5.5 kg (range: 0–10) in non-smokers with continuous abstinence compared to 3 kg (range: -5–8.5) for patients with at least 1 relapse (n=3). Median time for relapse was 121 days (range: 60–184) with 45 days (range: 3–120) of consumption during the 6 months period. Relapsed patients reported an actual low FTND score of 2.5 (range: 1–4) and high scores for motivation (median: 9; range: 8–10) and self-efficacy (10).

**Table 1:** Basic characteristics of study participants

*Characteristics*	*Mean*	*Range*
Age (years)	47.8	28–65
Age at start of smoking (years)	17.5	13–30
Years of smoking	31.3	11–49
Daily number of cigarettes	19.9	13–30
FTND score	6.5	4–8
Motivation to quit*	8.9	6–10
Self-efficacy*	7.5	3–10

* Scores for motivation to quit and self-efficacy were assessed on a self-reported scale from 1 (lowest) to 10. FTND: Fagerström test for nicotine dependence.

## DISCUSSION

This study evaluated the effects of a residential group behavior therapy program in CD patients admitted to a hospital solely for the purpose of smoking cessation.

In a cross-sectional survey of outpatients, Hilsden et al.^10^ found that CD patients are no more refractory to smoking cessation compared to the general population. However, studies evaluating the quit rates of smoking cessation therapy in CD patients are rare. In a large prospective Spanish multicenter study for smoking cessation Nunes et al.^2^ reported cessation rates of 31% (for patients being smoke-free for at least 1 week) with 23% continuous abstinence within a median follow-up of 9 months mostly after physicians’ advice and counseling. Cosnes et al.^11^ reported quit rates of 25% (patients who stopped smoking for more than 15 days) and 12% for patients remaining abstinent for more than 1 year.

Although reported quit rates in outpatient settings in CD patients are comparable to quit rates in the general population, there are numerous publications which suggest a better outcome in a residential setting exclusively for smoking cessation^5-7,12^. No data have been published as to quit rates specifically in CD patients resulting from a strictly residential smoking cessation program.

In this study, we report quit rates by residential cessation therapy of CD patients with a 72.7% continuous abstinence and 81.8% 7-day point prevalence abstinence. These results show that a residential smoking cessation program for CD patients is feasible and may be very effective.

Quit rates were at 100% at the end of the therapy assessed by CO-testing and during hospital treatment on a daily basis. Since participants were recruited nationwide, some of the participants travelled long distances (>800 km) for the treatment itself, therefore CO controls were not feasible for the follow-up at 6 months on-site for biovalidation. However, specifically in CD patients, Nunes et al.^2^ reported a good correlation between self-reported smoking habits and urinary cotinine and exhaled CO levels. Furthermore, all (n=11) of the patients reported having contacted at least one of the other participants of the study during the 6 months post-therapy period, some of them being in contact with other participants on several occasions. Although not specifically evaluated at the follow-up at 6 months, participants themselves spontaneously reported, to their knowledge, a very high quit rate within the group, consistent with our results.

Surprisingly, none of the patients tried to quit smoking with prescription medication. In the TABACHRON study most of the CD patients did not use either pharmacological support or NRT. The authors suggested that, in Spain, anti-tobacco therapies are expensive and not covered by the public health system, which could partly account for the low level of drug use in this population^2^. In contrast, in our study most (9/11) of the patients used NRT therapy, which also is not covered by the German public health system. Although gastrointestinal adverse effects of varenicline are common they are usually mild, such as nausea, constipation, indigestion or flatulence^13^ but may still be a reason for discontinuation in CD patients^14^. However, patients reported they were most concerned that the oral prescription medication might affect the course of the CD and decided to stay with NRT rather than take any oral prescription medication.

### Limitations

There are limitations to this study. First of all, it is a non-controlled study with low numbers of participants. In Germany, standard behavioral group therapy for smoking cessation is conducted in an outpatient setting on a weekly basis over the course of usually three to eight weeks. Therapy costs for smoking cessation are not covered by health insurances, although many insurances offer financial support for at least a fraction of the cost for outpatient treatment. Residential smoking cessation treatment in Germany is only available in few hospitals as an add-on when patients are admitted for treatment of specific illnesses, such as pulmonary or cardiac diseases but not exclusively for smoking-cessation. Therefore, it was not possible to include regular inpatients in this study.

## CONCLUSIONS

Residential treatment programs exclusively for smoking cessation are very effective^5-7,12^. This study shows that CD patients can also profit from a residential cessation therapy and promises high quit rates in this specific group of patients. These are results from a pilot study. For healthy smokers, results from a larger prospective, randomized and controlled study will be available in late 2022^15^. These two studies will provide a good basis for implementing a larger, controlled study in CD patients in the near future.

## Data Availability

The data supporting this research are available from the authors on reasonable request.
